# Selective serotonin reuptake inhibitors and suicidal behaviour: a population-based cohort study

**DOI:** 10.1038/s41386-021-01179-z

**Published:** 2021-09-24

**Authors:** Tyra Lagerberg, Seena Fazel, Arvid Sjölander, Clara Hellner, Paul Lichtenstein, Zheng Chang

**Affiliations:** 1 Department of Medical Epidemiology and Biostatistics, Karolinska Institutet, Stockholm, Sweden; 2 Department of Psychiatry, Warneford Hospital, University of Oxford, Oxford, UK; 3 Centre for Psychiatry Research, Department of Clinical Neuroscience, Karolinska Institutet, Stockholm, Sweden; 4 Stockholm Health Care Services, Stockholm County Council, Stockholm, Sweden

**Keywords:** Risk factors, Outcomes research

## Abstract

There is concern that selective serotonin reuptake inhibitor (SSRI) treatment may increase the risk of suicide attempts or deaths, particularly among children and adolescents. However, debate remains regarding the nature of the relationship. Using nationwide Swedish registers, we identified all individuals aged 6–59 years with an incident SSRI dispensation (*N* = 538,577) from 2006 to 2013. To account for selection into treatment, we used a within-individual design to compare the risk of suicide attempts or deaths (suicidal behaviour) in time periods before and after SSRI-treatment initiation. Within-individual incidence rate ratios (IRRs) of suicidal behaviour were estimated. The 30 days before SSRI-treatment initiation was associated with the highest risk of suicidal behaviour compared with the 30 days 1 year before SSRI initiation (IRR = 7.35, 95% CI 6.60–8.18). Compared with the 30 days before SSRI initiation, treatment periods after initiation had a reduced risk—the IRR in the 30 days after initiation was 0.62 (95% CI 0.58–0.65). The risk then declined over treatment time. These patterns were similar across age strata, and when stratifying on history of suicide attempts. Initiation with escitalopram was associated with the greatest risk reduction, though CIs for the IRRs of the different SSRI types were overlapping. The results do not suggest that SSRI-treatment increases the risk for suicidal behaviour in either youths or adults; rather, it may reduce the risk. Further research with different study designs and in different populations is warranted.

## Introduction

Suicide is the tenth leading cause of death in North America, and the leading cause in those aged 15–24 years worldwide [1]. For every adult who dies by suicide, more than 20 others attempt it [2]. Among the modifiable risk factors for suicidal behaviour are mood disorders [3], for which antidepressant medications are the main pharmacological treatment option [4, 5]. Selective Serotonin Reuptake Inhibitors (SSRIs) are the most prevalent type of antidepressant in most countries [4, 5], and typically the first-line pharmacological treatment option for mood disorders [6, 7].

Despite indications that SSRIs may improve core depressed mood symptoms [8], concerns have been raised about the risk of suicide attempts and deaths (suicidal behaviour) caused by SSRIs, particularly among the young. Randomised controlled trials (RCTs) have found evidence of an increased risk of newly onset suicidal ideation and behaviour among children and adolescents in SSRI-treatment arms [9], though the risk among adults is less clear [10–13].

However, RCTs tend to have short follow-ups and limited generalisability to real-world settings, and individual RCTs are under-powered for rarer outcomes such as suicide attempt and completion. They also routinely exclude high-risk groups, such as individuals with a history of suicidal ideation or attempts [14, 15], and people with common comorbidities, such as substance misuse. Observational studies can therefore provide valuable evidence from representative populations with longer follow-up. Previous studies using these designs have generally found an association between SSRI use and suicidal behaviour in children and adolescents [16, 17], with some conflicting evidence among adults [18–20]. There is also observational evidence that early phases of antidepressant treatment may carry particularly high risks of suicidal behaviour [19].

However, previous observational studies are subject to bias from unmeasured confounding—particularly confounding by indication, which is when the characteristics that indicate an individual for antidepressant treatment also predispose them to the outcome under investigation. For example, a recent umbrella review of observational studies found strong evidence of an association between SSRI use and suicidal behaviour in children and adolescents, but concluded that the association was likely to be biased by confounding by indication [20]. The fact that some patients are already at risk of suicidal behaviour before SSRI initiation due to the disorder indicating them to treatment must therefore be taken into account when assessing the safety profile of these drugs.

The present study examines the risk of suicidal behaviour in periods around SSRI-treatment initiation, in a national cohort of SSRI new users. We assess the risk of suicidal behaviour up to 1 year before and after initiation of an SSRI, as well as the risk before and after any SSRI-treatment start over a follow-up of up to 7.5 years. We also consider the impact of age and a history of previous suicide attempts. The null hypothesis was that there was no association between SSRI-treatment initiation and suicidal behaviour, including in different age groups.

## Materials and methods

### Data sources

We linked information from several Swedish national registers using unique personal identification numbers [21]. Prescription information was extracted from the Swedish Prescribed Drug Register, which documents all dispensed pharmaceuticals in Sweden since July 2005 [22]. Demographic information was extracted from the Total Population Register [23], and emigration data from the Migration Register [23]. The Cause of Death Register was used to extract information on dates and causes of death [24]. The National Patient Register provided information on inpatient care since 1973 and non-general practitioner outpatient care since 2001, including discharge diagnoses [25]. The study was approved by the Regional Ethics Committee (Stockholm, Sweden). Informed consent is not required for register-based studies in Sweden [26].

### Cohort

We identified individuals aged 6–59 years that collected their initiating SSRI prescription during July 1st 2006 to December 31st 2013. Initiating dispensations were defined as an SSRI dispensation preceded by at least 365 days without any SSRI prescription dispensations. As the Swedish PDR records prescriptions from July 2005, all included individuals had at least a 1-year period free of SSRI dispensations before their initiating dispensation. Individuals who had an emigration record prior to their initiating dispensation were excluded.

**Fig. 1:**
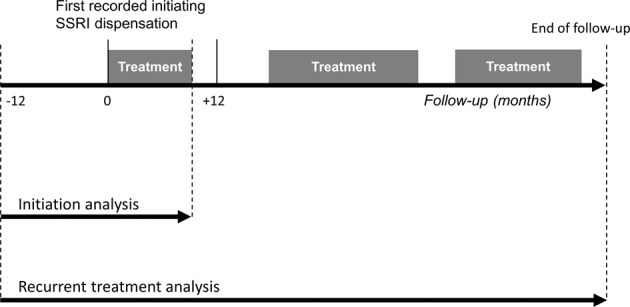
Illustration of study design for the initiation and recurrent treatment analyses. The dashed lines represent start and end of the time periods considered in the initiation and recurrent treatment analyses.

To investigate the risk of suicidal behaviour around the first recorded treatment initiation (‘initiation analysis’), we considered the time period 1 year before and up to 1 year after treatment initiation. For these analyses, follow-up ended at date of emigration, death, an individual’s 60th birthday, 1 year after treatment initiation, or date of SSRI-treatment end, whichever occurred first (Fig. 1).

To assess the risk of suicidal behaviour over recurrent treatment, we also considered a longer follow-up where individuals could go on and off SSRI-treatment multiple times (‘recurrent treatment analysis’). For these analyses, follow-up started 1 year prior to the dispensation date of the initiating prescription, and ended at date of first emigration, death, reaching age 60, or 31st December 2013, whichever occurred first (Fig. 1).

### Measures

#### Exposure

The exposure of interest was SSRI medication (ATC code: N06AB). The SSRIs included were all those that were licensed for sale in Sweden during the study period: fluoxetine, citalopram, paroxetine, sertraline, fluvoxamine, and escitalopram. We have focused on SSRIs, as they are the recommended first-line pharmacological treatment for depression according to Swedish guidelines [27]. They are also the most commonly used antidepressant in Sweden—by a wide margin in the case of individuals aged below 18 years [28]. A treatment period started at the dispensation date of a prescription. Two dispensations falling within 120 days (4 months) of each other were considered to belong to the same treatment period [29]. At the last or single dispensation in a treatment period, the treatment end was defined by adding the population average number of days between consecutive dispensations for the specific medication type to the date of dispensation.

In a sensitivity analysis, we used an alternative definition of SSRI-treatment periods based on the assumption that individuals take one SSRI pill per day. Using this assumption, we estimated the treatment length from the number of pills included in each dispensation.

#### Outcomes

The outcome was suicidal behaviour, which included hospital visits (outpatient attendance or inpatient admission) for suicide attempts, and deaths from suicide. In line with previous studies [30], we included events with both known intent (ICD-10 codes X60–X84) and unknown intent (ICD-10 codes Y10–Y34). In a sensitivity analysis, we included only outcomes with known intent.

#### Covariates

All analyses were adjusted for time-varying treatment with non-SSRI antidepressants (N06A, excluding N06AB), benzodiazepines (ATC code: N05BA), and any other psychotropic medications (all binary covariates; see Table S1 for ATC-codes for other psychotropic medications). Recurrent treatment analyses were additionally adjusted for age (continuous variable).

### Analyses

All analyses were carried out using conditional Poisson regression to estimate within-individual incidence rate ratios (IRRs). The comparison between treated and untreated time periods were made within individuals, by using each individual as his or her own control, thereby controlling for all confounding factors that are stable within a person over time, even if they are unmeasured [31]. Analyses were carried out overall and as stratified by age group (6–17 year olds, 18–24 year olds, 25–39 year olds, 40–49 year olds, and 50–59 year olds).

The initiation analysis considered the year before and up to 1 year after the first recorded SSRI dispensation. IRRs were estimated in two models: one where the month 1 year prior to initiation (month −12) was the reference category; the other where the month immediately prior to initiation (month −1) was the reference category. Throughout analyses, 1 month was considered to consist of 30 days. We also investigated the risk of suicidal behaviour around SSRI-treatment start when stratifying on a history of suicide attempts prior to 1 month before treatment initiation. Further, we examined the associations in individuals with specific disorders: Attention-Deficit Hyperactivity Disorder (ADHD), Autism Spectrum Disorder (ASD), Bipolar Disorder (BPD), and Substance Use Disorder (SUD) prior to 1 month before treatment initiation (see Table S2 for ICD codes). We considered these disorders as they are associated with suicide risk in themselves and because they are relatively common, making them good candidates for assessing whether the association between SSRI initiation and suicidal behaviour is influenced by different comorbidities. We also conducted analyses by the different SSRI types given on the date of initiation—in the overall cohort and in individuals aged 6–17 years. We conducted sensitivity analyses using alternative definitions for the outcome and exposure. To assess the possible impact of unmeasured confounding, we calculated the E-value for the effect estimate in the month immediately after initiation compared to immediately prior to initiation in the overall cohort [32].

In the recurrent treatment analyses, considering all SSRI-treatment periods during the full follow-up, we subdivided the on-treatment time to consider the risk of suicidal behaviour in the periods 30 days, 31–120 days, and more than 120 days after treatment start. Off-treatment periods were subdivided into 30 days immediately prior to the next treatment start, and periods more than 30 days before the next treatment start (reference category) (Fig. 1).

Throughout analyses, p-values at or below 0.05 were considered to denote statistical significance. All statistical tests were two-tailed. The data management was carried out in SAS version 9.4. Statistical analyses and figures were generated using R version 3.6.3.

## Results

We identified 538,577 individuals who had their first recorded SSRI dispensation during July 1st 2006 to December 31st 2013 (see Fig. S1 for a flowchart of cohort inclusion). Most were female (62.4%, *N* = 336,032; Table 1). Individuals aged 25–39 years made up the largest proportion (34.6%, *N* = 186,571). A majority of individuals or their parents had commenced either upper secondary school or university as their highest level of education (45.9% and 43.5%, respectively). In the year before initiation, suicide attempts occurred in 1.3% (*N* = 6745) of individuals. In the year after, suicide attempts occurred in 1.2% (*N* = 6080) of individuals, and death by suicide in 0.1% (*N* = 346). Over the full follow-up (mean 4.6 years), suicide attempts occurred in 4.4% (*N* = 23,934) of individuals, and suicide deaths in 0.3% (*N* = 1703). The rate of suicide death in our study was approximately 68.7 per 100,000 person-years. This is higher than the crude rate of suicide death in the general Swedish population in 2013 (15.9) [33], as expected. Sample characteristics were similar across females and males, though males had a higher percentage of individuals who died by suicide (0.5% versus 0.2%). At initiation, most individuals received Citalopram (*N* = 207,659; 38.6%), followed by Sertraline (*N* = 200,810; 37.3%) (Fig. S2).

**Table 1:** Sample characteristics of the study cohort.

	Overall (*N* = 538,577)		Female (*N* = 336,032)		Male (*N* = 202,545)	
No. individuals at different ages^a^ (%)						
6–17 years	31,280	(5.8 %)	19,533	(5.8%)	11,747	(5.8%)
18–24 years	91,365	(17.0%)	58,415	(17.4%)	32,950	(16.3%)
25–39 years	186,571	(34.6%)	117,453	(35.0%)	69,118	(34.1%)
40–49 years	123,795	(23.0%)	77,476	(23.1%)	46,319	(22.9%)
50–59 years	105,566	(19.6%)	63,155	(18.8%)	42,411	(20.9%)
No. individuals at different education^b^ levels (%)						
Compulsory school (≤9 years)	47,887	(8.9%)	27,125	(8.1%)	20,762	(10.3%)
Upper secondary school	247,278	(45.9%)	150,786	(44.9%)	96,492	(47.6%)
University	234,399	(43.5%)	152,767	(45.5%)	81,632	(40.3%)
No information	9013	(1.7%)	5354	(1.6%)	3659	(1.8%)
Mean length of full follow-up, yrs	4.6		4.7		4.6	
No. individuals with event (%)						
Year before initiation	6745	(1.3%)	4169	(1.2%)	2576	(1.3%)
Suicide attempts	6745	(1.3%)	4169	(1.2%)	2576	(1.3%)
Year after initiation	6385	(1.2%)	4311	(1.3%)	2074	(1.0%)
Suicide attempts	6080	(1.2%)	4222	(1.3%)	1858	(1.0%)
Death by suicide	346	(0.1%)	104	(<0.1%)	242	(0.1%)
Full follow-up	25,090	(4.7%)	15,455	(4.6%)	9635	(4.8%)
Suicide attempts	23,934	(4.4%)	15,087	(4.5%)	8847	(4.4%)
Death by suicide	1703	(0.3%)	601	(0.2%)	1102	(0.5%)

^a^At first SSRI treatment initiation.

^b^Highest level of commenced education between the index individual and his/her parents in the calendar year before the year of initiation.

### Initiation analysis

**Fig. 2:**
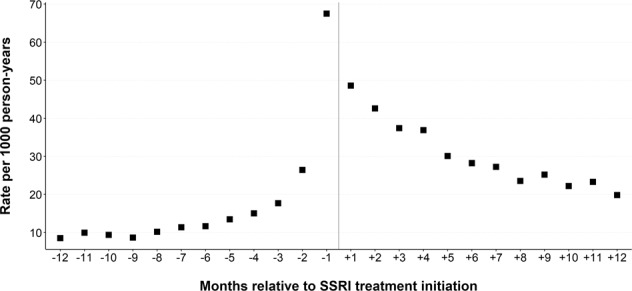
Rate of suicidal behaviour in months relative to first SSRI initiation. Rate per 1000 person-years by month in the year prior to (month −12 to −1) and after (month +1 to +12) first SSRI initiation.

Fig. 2 presents the absolute rate of suicidal behaviour in the year prior to and following the first recorded initiating dispensation. The rate peaked in the month immediately prior to initiation, at 67.5/1000 person-years. In the month following initiation, the rate fell to 48.6/1000 person-years, after which there was a relatively steady decline to 19.8/1000 person-years in month 12 after initiation.

**Fig. 3:**
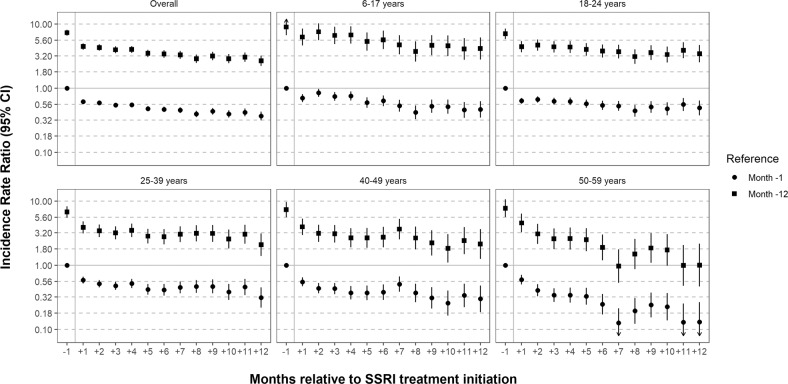
Within-individual incidence rate ratios of suicidal behaviour in months relative to first SSRI initiation. Circles represent analyses where the reference period is month −1 of the follow-up; squares represent analyses where the reference period is month −12 of the follow-up.

Figure 3 shows within-individual IRRs in two models (see IRR estimates in Table S3). When using month −12 as reference, incidence rates for month −1 through +12 were all elevated and statistically significant. The IRR was the highest in month −1 (7.35, 95% CI, 6.60–8.18 in the overall cohort). It then gradually declined—from an IRR of 4.47 in month +1 to an IRR of 2.68 in month +12 in the overall cohort. In this model, 1.6% of individuals (*N* = 8833) were informative by virtue of having at least one event during month −12, or during months −1 through +12. When using month −1 as reference (in this model, 8560 individuals were informative), all months following treatment initiation had statistically significantly lowered incidence rates, with a gradual decline over time (from an IRR of 0.62 in month +1 to one of 0.37 in month +12 in the overall cohort). These patterns were relatively consistent across age and sex strata (Tables S3, S4). The strongest measured time-varying confounder was benzodiazepine treatment (IRR = 1.71, 95% CI, 1.58–1.86; Table S1). The E-value necessary to negate a causal interpretation of the IRR in month +1 versus month −1 in the overall cohort was 2.61 (given our observed point estimate of 0.62; Fig. S3). For the upper confidence limit (0.65), it was 2.45 [32].

A total of 4.2% (*N* = 22,396) of the cohort had a history of suicide attempts before month −1. Stratifying on this factor did not substantially affect within-individual IRR estimates overall or by age (Table S5). However, the crude absolute rates per 1000 person-years differed substantially by history of suicide attempts (Fig. S4). The rate of events in month +1 among those with a history of suicide attempts was about 8 times higher than among those without (302/1000 person-years versus 38/1000 person-years).

In individuals with prior diagnoses of ADHD, ASD, and BPD diagnoses, most IRR estimates were non-significant (Table S2). For individuals with SUD (*N* = 34,331; 6.4%), SSRI was associated with similar risk reduction as in the overall cohort.

The results were also broadly consistent across common SSRI types at initiation. Escitalopram- and fluoxetine-treated individuals had the lowest IRR in the overall cohort and 6–17 year olds, respectively (IRRs of 0.53 and 0.65 in the month immediately after initiation; Figs. S2, S5).

In sensitivity analyses using alternative outcome and exposure definitions, the results were similar to the main analysis (Table S6).

### Recurrent treatment analysis

In the analysis of recurrent treatment periods (Table 2), 25,090 individuals were informative by virtue of having had at least one event during the full follow-up. In the overall cohort, the period within 30 days before treatment start had the highest IRR (3.25, 95% CI, 3.15–3.36). There was then a gradual decline—from 2.07 (95% CI, 2.00–2.15) in the first 30 treated days after initiation, to 1.55 (95% CI, 1.50–1.59) in the treated period more than 120 days after initiation. This pattern was relatively consistent across age strata.

**Table 2:** Within-individual incidence rate ratio of suicidal behaviour in time periods relative to treatment initiation in the recurrent treatment analysis.

		Before treatment initiation		After treatment initiation		
Age		>30 days	0–30 days	0–30 days	31–120 days	>120 days
Overall	No. events	29,774	4559	3479	6485	7941
	IRR (95% CI)	1	3.25 (3.15–3.36)	2.07 (2.00–2.15)	1.97 (1.91–2.03)	1.55 (1.50–1.59)
6–17 years	No. events	2752	677	517	1231	1394
	IRR (95% CI)	1	3.57 (3.28–3.90)	2.52 (2.29–2.79)	2.79 (2.59–3.00)	2.07 (1.91–2.25)
18–24 years	No. events	10,002	1419	1189	2342	2855
	IRR (95% CI)	1	3.05 (2.88–3.23)	2.02 (1.89–2.15)	2.06 (1.96–2.17)	1.69 (1.6–1.78)
25–39 years	No. events	9783	1235	919	1640	2081
	IRR (95% CI)	1	2.80 (2.63–2.98)	1.73 (1.61–1.86)	1.60 (1.51–1.7)	1.30 (1.22–1.38)
40–49 years	No. events	4353	719	474	762	1033
	IRR (95% CI)	1	3.36 (3.09–3.65)	1.94 (1.76–2.15)	1.63 (1.50–1.77)	1.44 (1.33–1.57)
50–59 years	No. events	2884	509	380	510	578
	IRR (95% CI)	1	3.28 (2.97–3.62)	2.18 (1.95–2.45)	1.52 (1.37–1.68)	1.05 (0.94–1.17)

## Discussion

In this population-based cohort study of 538,577 individuals who initiated SSRI treatment, we found that the risk for suicidal behaviour was highest in the month immediately preceding the first recorded SSRI initiation. On-treatment periods following initiation were associated with a lower risk compared to the month immediately prior to initiation, with the risk then gradually decreasing over time. However, the risk during on-treatment periods remained elevated as compared to the off-treatment period 1 year before treatment start. This pattern was consistent across age groups, when stratifying on a history of suicide attempts, and in analyses of recurrent treatment periods.

Prior observational studies that investigate the risk of suicidal behaviour around antidepressant treatment initiation have generally found a similar pattern of results to ours—i.e. that risk is highest immediately prior to treatment initiation [34–37]. However, given a lack of direct comparisons between periods before and after initiation [34, 35], or low power to investigate the associations by age [36, 37] and/or sex [36], the change in risk following initiation remains unclear, particularly among important clinical subgroups.

While we find that the risk of suicidal behaviour was highest immediately prior to initiation and that the risk then declines over treatment time, we found that risk during on-treatment periods remained elevated compared to the month 1 year before treatment initiation. This suggests that, in the first year of treatment, SSRI medication does not bring the risk of suicidal behaviour to baseline, which may be related to findings from previous studies that around 30% of individuals do not achieve remission from depression within 12 months of antidepressant treatment [38].

Importantly, we found that associations were not substantially different across age categories. However, we cannot rule out the possibility of an age effect, given that the impact of unmeasured confounders may be different across the different age categories. In Sweden, most individuals under the age of 18 years receive psychiatric care from specialist services, while adults routinely receive it from primary care. This means younger individuals may receive different clinical monitoring and/or other attention from the healthcare service in addition to their SSRI medication.

Our finding that a history of suicide attempts is associated with a higher absolute rate of suicidal behaviour is consistent with studies showing that past suicide attempts is a predisposing factor for completed suicide [1] and a predictor of future attempts [39]. We found no evidence that diagnosis with ADHD, ASD, BPD, or SUD influenced the impact of SSRI initiation on the risk of suicide. In addition, our findings were similar when considering recurrent treatment over the full follow-up, suggesting that SSRI treatment may reduce the risk of suicidal behaviour regardless of whether the individual has received treatment previously or not.

Our results suggest that clinicians should be reassured that, overall, prescribing SSRI medications is not associated with an increased risk of suicidal behaviour when comparing periods immediately following initiation to periods immediately prior to it. However, we cannot claim causality or exclude the possibility that—among individuals with certain risk profiles—SSRI initiation may precipitate onset of suicidal behaviour. The risk may also remain elevated for up to 1 year after treatment initiation compared to 1 year prior to initiation, possibly due to residual risk from the underlying disorder indicating an individual for treatment. Our findings were similar in both initiation analyses and recurrent treatment analyses, suggesting that clinicians be vigilant of a residual risk regardless of whether the treated individual has taken SSRIs previously or not. With regard to individual SSRIs, the ranking of the effect estimates corresponds to the ranking of antidepressant efficacy from a network meta-analysis of trial data [40], with escitalopram associated with the largest reduction in the risk of suicidal behaviour. However, we could not draw strong conclusions from the analyses on individual SSRIs, given the overlapping confidence intervals and that we could not account for the factors selecting individuals into treatment with different SSRI types. Absolute rates of suicidal behaviour were highest in individuals with a history of suicide attempts, suggesting that these patients should be the focus of clinical attention. Identification of further high-risk groups could be aided by prognostic models [41]. Finally, among other psychotropic medications, we found that benzodiazepine treatment was associated with the strongest increased risk of suicidal behaviour independently of SSRIs. This corresponds to prior evidence from RCTs and observational studies that benzodiazepine treatment is associated with an increased risk for suicide [42], though further investigation is warranted.

### Strengths and limitations

One key strength of this study is that it draws on a representative nationwide cohort. To our knowledge, our analyses of the risk of suicidal behaviour contain the highest number of SSRI initiators to date. Moreover, the within-individual comparison ensured that we controlled for all unmeasured confounders that are stable over time in a person.

However, there are several limitations. First, as in any observational research, unmeasured confounding is expected to affect our estimates, meaning we cannot claim that our results are causal. While our design inherently controlled for all time-stable confounding, it is possible that unmeasured time-varying factors, e.g. related to healthcare access, could lead to our observed association between SSRI initiation and risk of suicidal behaviour. These factors could include psychotherapeutic interventions, which may reduce the risk for suicide [43]. If an individual receives their SSRI prescription in inpatient care, the fact that a patient is in a hospital could also lead to lower suicide risk, due to greater clinical monitoring and reduced access to means by which to carry out suicidal behaviour. Specifically, this limitation could apply if a suicide attempt in the period preceding SSRI initiation leads to an inpatient stay, and if a patient initiates the SSRI during their time in hospital. The E-value is a metric suggested for quantifying the amount of unmeasured confounding necessary to negate a causal interpretation of an effect estimate [32]. In our comparison of month +1 with month −1 in the overall cohort, the E-value would be 2.61 for the point estimate (0.62), and 2.45 for the upper confidence limit (0.65). These E-values are moderate, indicating that the unmeasured confounding necessary to negate a causal interpretation would have to have a larger effect than our strongest measured confounder (benzodiazepine treatment). However, the E-value likely reflects the combined effects of more than one unmeasured confounder [44]. We therefore do not have strong grounds to claim that the risk reduction immediately following SSRI initiation is causal. Second, we used data from the National Patient Register to identify suicide attempts, meaning we could not capture attempts that did not lead to healthcare contact. We also could not consider suicidal ideation, which is an important outcome in RCTs. On the other hand, our measure of suicidal behaviour likely reflects the more severe suicide attempts that carry the greatest cost to individuals and society. Third, it is possible that our treatment definition leads to some misclassification of treatment duration, as the Swedish PDR does not include intended days of supply. However, a sensitivity analysis using an alternative treatment definition did not lead to substantially different results. Fourth, we do not know whether purchased medications were consumed, though this is true of any observational study. Fifth, we do not have information on indications for SSRI treatment, which precludes a more detailed analysis of whether the risk prior to initiation differs by the disorder indicating an individual for treatment. Sixth, death by suicide was rare in our cohort, and our within-individual study design meant we could not include individuals who died by suicide before they had time to initiate SSRIs. This means our results mainly pertain to suicide attempt. However, suicide attempt is a serious outcome, and may also predispose individuals to future completed suicide [45]. Finally, our results derive from a cohort representative of the Swedish population during the study period, but are not necessarily generalisable to other countries or time periods.

## Conclusion

Our results do not indicate that SSRI treatment increases the risk of suicidal behaviour; it is possible that SSRI treatment reduces the risk. However, individuals may remain at higher risk of suicidal behaviour at least throughout the first year of SSRI treatment relative to 1 year prior to initiation, which could be related to the underlying indication for treatment. The results suggest that, overall, clinicians should be reassured that SSRI initiation is not associated with an increased risk of suicidal behaviour. We cannot claim that our findings are causal, exclude the possibility of an age effect, or discount the possibility that SSRI treatment could precipitate suicidal behaviour in individuals with certain risk profiles.

## Supplementary information

Supplemental material
