# Supplementary material for: Selective serotonin reuptake inhibitors and suicidal behaviour: a population-based cohort study
Source: Neuropsychopharmacology. 2021 Sep 24;47(4):817–23. doi: 10.1038/s41386-021-01179-z (PMC8882171; doi:10.1038/s41386-021-01179-z)
Supplement: Supplementary file 1 — Supplemental material [file 41386_2021_1179_MOESM1_ESM.docx]

**SUPPLEMENT**

**Contents**

[**Figure S1. Flowchart of cohort inclusion** 2](#_Toc64995825)

[**Figure S2. Within-individual incidence rate ratios in month +1 in overall cohort (reference category: month -1), by SSRI type given at treatment initiation** 3](#_Toc64995826)

[**Figure S3. Distribution of IRR_EU_^a^ and IRR_UD_^b^ given different assumptions on the true effect of SSRI initiation on suicidal behaviour** 4](#_Toc64995827)

[**Figure S4. Rate of suicidal behaviour per 1000 person-years in months relative to first SSRI initiation, stratified by history of suicide attempts.** 5](#_Toc64995828)

[**Figure S5. Within-individual incidence rate ratios in month +1 in 6-17-year-olds (reference category: month -1), by SSRI type given at treatment initiation** 6](#_Toc64995829)

[**Table S1. Within-individual incidence rate ratio estimate of measured time-varying confounders in the overall cohort when month -1 is used as the reference category in the initiation analysis** 7](#_Toc64995830)

[**Table S2. Within-individual incidence rate ratio of suicidal behaviour in months relative to first SSRI initiation, by diagnosis** 8](#_Toc64995831)

[**Table S3. Within-individual incident rate ratio of suicidal behaviour in months relative to first SSRI initiation** 9](#_Toc64995832)

[**Table S4. Within-individual incidence rate ratio of suicidal behaviour in months relative to first SSRI initiation, stratified by sex** 10](#_Toc64995833)

[**Table S5. Within-individual incidence rate ratio of suicidal behaviour relative to first SSRI initiation, stratified by history of suicide attempts and age** 11](#_Toc64995834)

[**Table S6. Within-individual incidence rate ratios of suicidal behaviour in initiation analysis using alternative outcome and exposure definitions** 12](#_Toc64995835)


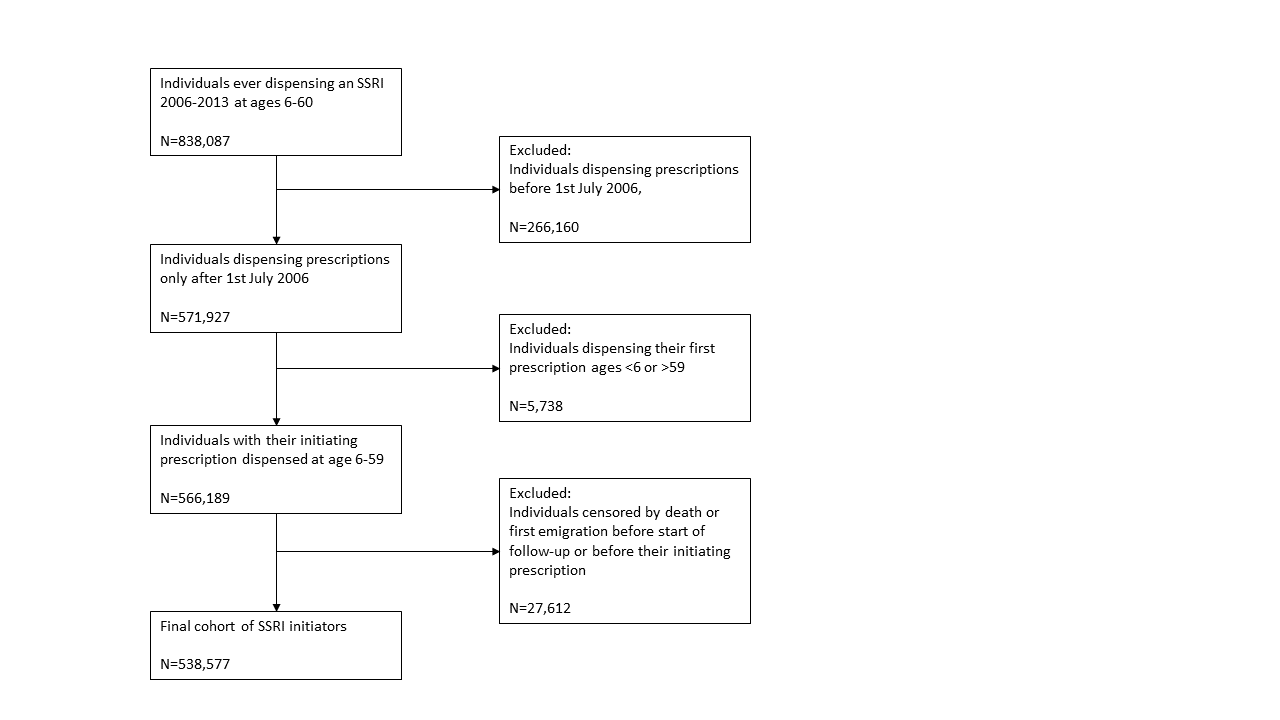


# **Figure S1. Flowchart of cohort inclusion**

# **Figure S2. Within-individual incidence rate ratios in month +1 in overall cohort (reference category: month -1), by SSRI type given at treatment initiation**


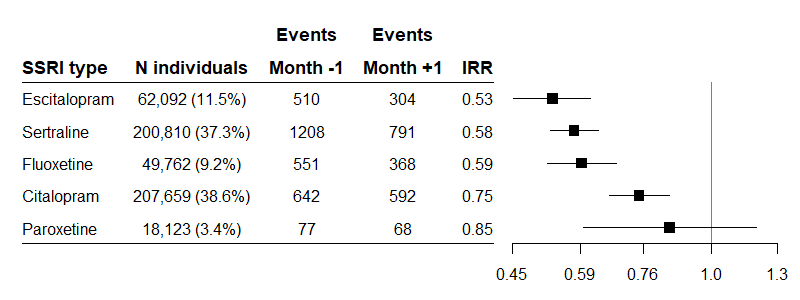


Fluvoxamine initiation (n=237, <0.1%) was not included, as there were too few events to estimate IRRs (0 events in month -1).

An individual could contribute to more than one SSRI type only if they were prescribed more than one type of SSRI on the date of SSRI initiation. This was the case for 106 individuals (<0.1%).

# **Figure S3. Distribution of IRR_EU_^a^ and IRR_UD_^b^ given different assumptions on the true effect of SSRI initiation on suicidal behaviour**

**
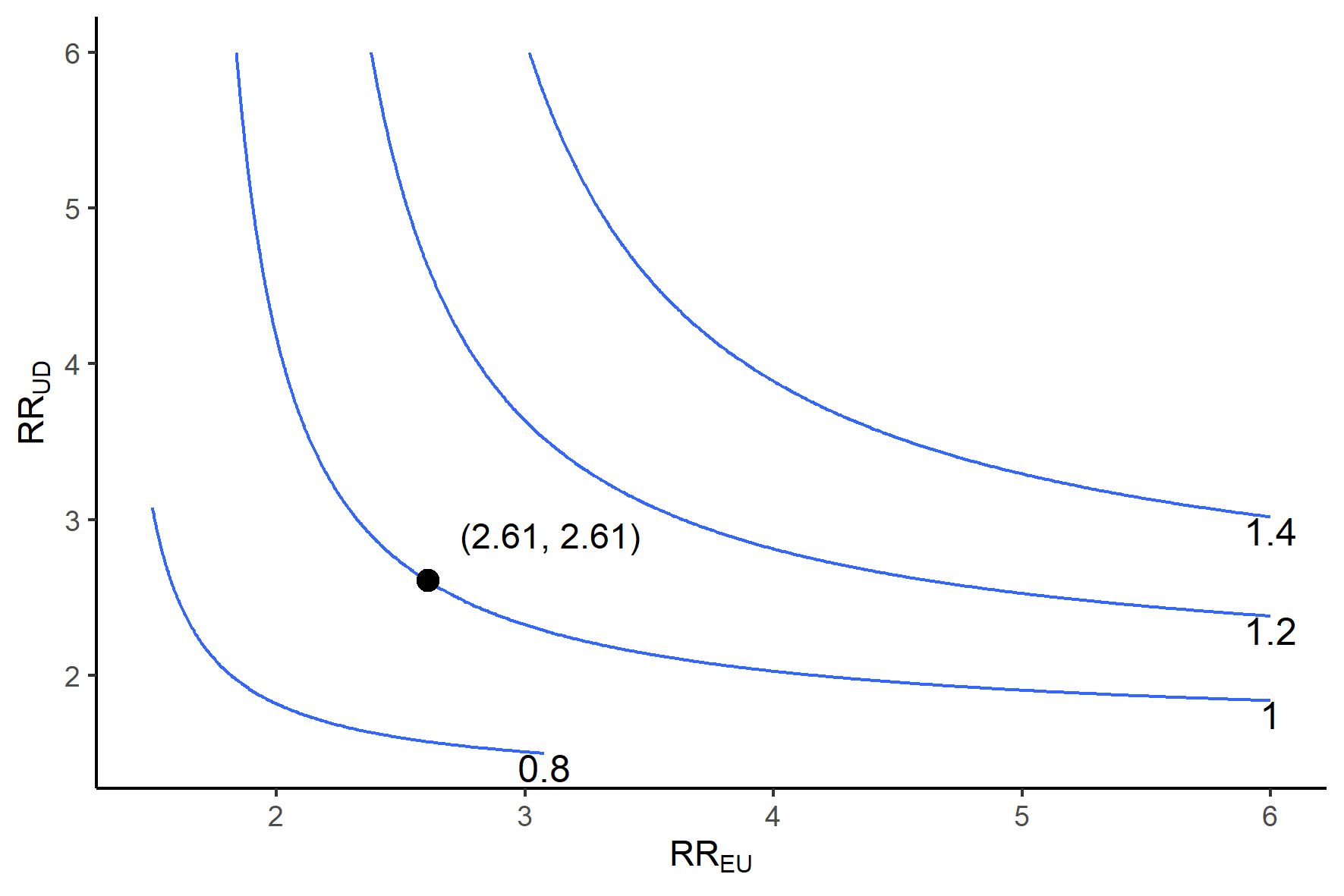
**

^a^ IRR_EU_: Association between unmeasured confounders and the exposure

^b^ IRR_UD_: Association between unmeasured confounders and the outcome

The different curves represent true effects of the exposure on the outcome. Given a specified true effect, the IRR_EU_ and IRR_UD_ can have any combination of values along the relevant curve

# **Figure S4. Rate of suicidal behaviour per 1000 person-years in months relative to first SSRI initiation, stratified by history of suicide attempts.**

**
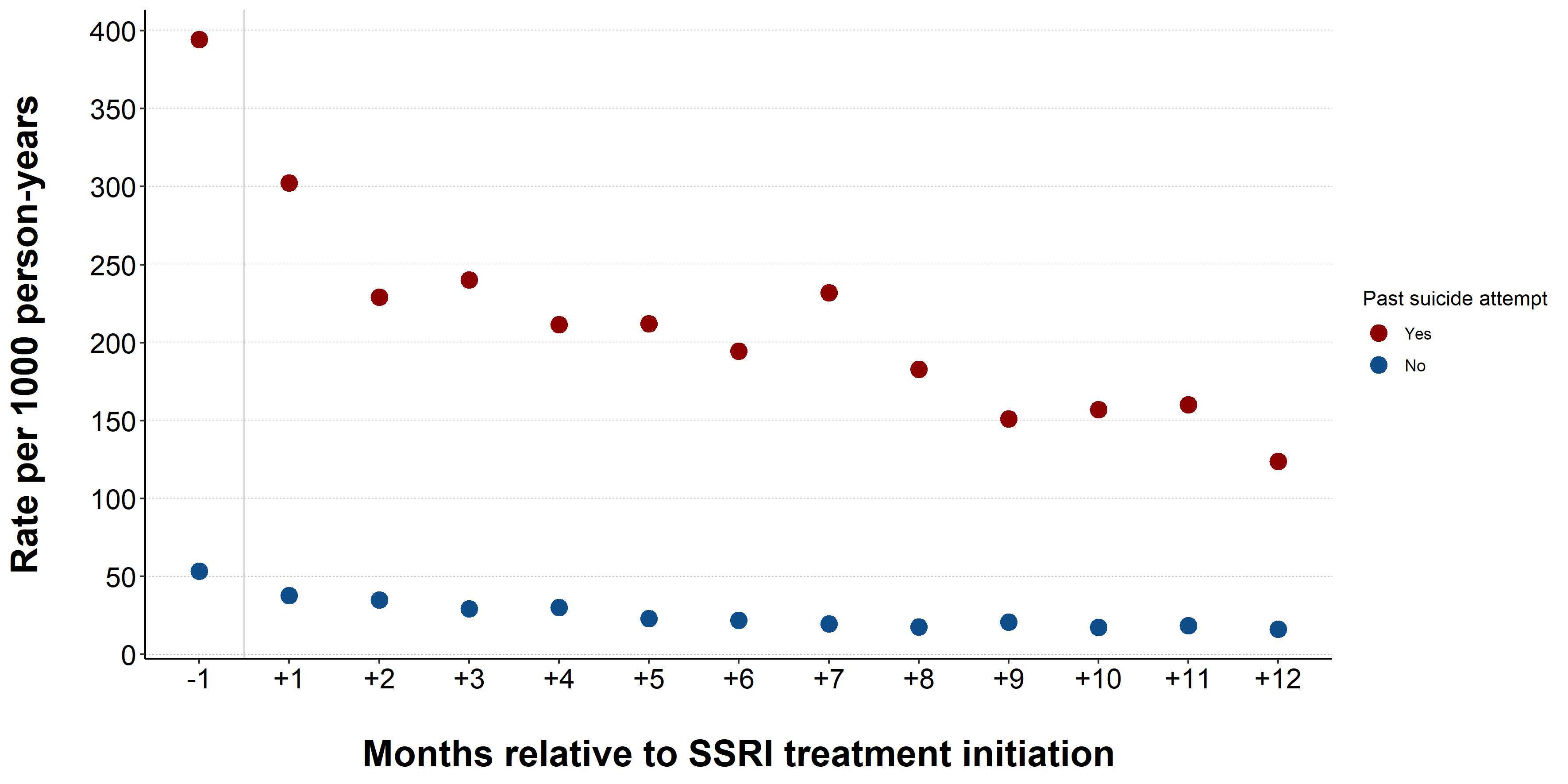
**

#
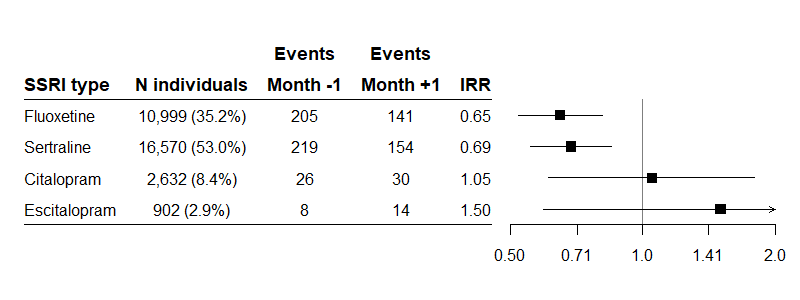
**Figure S5. Within-individual incidence rate ratios in month +1 in 6-17-year-olds (reference category: month -1), by SSRI type given at treatment initiation**

Note: Paroxetine and fluvoxamine initiation (n=162, 0.5%; n=19, <0.1%) were not included, as there were too few events to estimate IRRs (0 events in either month -1 or month +1).

An individual could contribute to more than one SSRI type only if they were prescribed more than one type of SSRI on the date of SSRI initiation. This was the case in 4 individuals (<0.1%).

# **Table S1. Within-individual incidence rate ratio estimate of measured time-varying confounders in the overall cohort when month -1 is used as the reference category in the initiation analysis**

| **Variable^a^** | **IRR (95% CI)** |
| --- | --- |
| Treatment with non-SSRI antidepressants | 1.19 (1.09-1.29) |
| Treatment with benzodiazepines | 1.71 (1.58-1.86) |
| Treatment with other psychotropic drugs | 1.37 (1.29-1.45) |
| ^a^ All time-varying treatment covariates are considered as binary variables.  Non-SSRI antidepressants (N06A, excluding N06AB)  Benzodiazepines (N05BA)  Other psychotropic drugs (N02A, N03A, N05A, N05B excluding N05BA, N05C, N06B, N06D, N07B) | |

# **Table S2. Within-individual incidence rate ratio of suicidal behaviour in months relative to first SSRI initiation, by diagnosis**

|  | **ADHD (N=10,057; 1.9%)** | | **ASD (N=5,095; 1.0%)** | | **BPD (N=4,749; 0.9%)** | | **SUD (N=34,331; 6.4%)** | |
| --- | --- | --- | --- | --- | --- | --- | --- | --- |
| **Month** | **No. Events** | **IRR (95% CI)** | **No. Events** | **IRR (95% CI)** | **No. Events** | **IRR (95% CI)** | **No. Events** | **IRR (95% CI)** |
| **-1** | 104 | 1 | 41 | 1 | 62 | 1 | 648 | 1 |
| **+1** | 98 | 0.95 (0.72-1.26) | 45 | 1.08 (0.70-1.65) | 59 | 1.05 (0.72-1.52) | 497 | 0.70 (0.62-0.79) |
| **+2** | 66 | 0.84 (0.61-1.16) | 23 | 0.76 (0.45-1.30) | 43 | 0.91 (0.60-1.37) | 308 | 0.57 (0.50-0.66) |
| **+3** | 63 | 0.96 (0.69-1.33) | 27 | 1.23 (0.73-2.06) | 19 | 0.54 (0.31-0.92) | 242 | 0.56 (0.48-0.66) |
| **+4** | 48 | 0.96 (0.67-1.38) | 21 | 1.24 (0.70-2.19) | 24 | 0.92 (0.55-1.52) | 199 | 0.64 (0.54-0.76) |
| **+5** | 32 | 0.83 (0.54-1.26) | 20 | 1.41 (0.79-2.53) | 17 | 0.80 (0.45-1.42) | 130 | 0.52 (0.42-0.63) |
| **+6** | 34 | 1.06 (0.70-1.61) | 27 | 2.12 (1.23-3.66) | 11 | 0.68 (0.34-1.33) | 114 | 0.55 (0.45-0.69) |
| **+7** | 21 | 0.76 (0.46-1.25) | 10 | 0.83 (0.40-1.74) | 15 | 1.13 (0.61-2.10) | 95 | 0.56 (0.44-0.70) |
| **+8** | 19 | 0.76 (0.45-1.28) | 5 | 0.46 (0.18-1.22) | 8 | 0.64 (0.29-1.40) | 65 | 0.45 (0.35-0.59) |
| **+9** | 14 | 0.71 (0.39-1.27) | 13 | 1.51 (0.76-3.00) | 9 | 1.03 (0.48-2.18) | 58 | 0.49 (0.37-0.65) |
| **+10** | 12 | 0.78 (0.42-1.47) | 9 | 1.27 (0.58-2.78) | 4 | 0.57 (0.20-1.63) | 51 | 0.52 (0.38-0.70) |
| **+11** | 8 | 0.61 (0.29-1.29) | 7 | 1.11 (0.47-2.62) | 9 | 1.42 (0.66-3.07) | 62 | 0.73 (0.55-0.97) |
| **+12** | 2 | 0.17 (0.04-0.71) | 9 | 1.64 (0.74-3.63) | 6 | 1.15 (0.47-2.84) | 36 | 0.50 (0.35-0.71) |
| All diagnoses before month -1 were considered.  The diagnosis groups are not mutually exclusive.  ADHD: Attention-Deficit Hyperactivity Disorder (ICD10-code: F90)  ASD: Autism Spectrum Disorder (ICD10-code: F84)  BPD: Bipolar Disorder (ICD10-code: F31)  SUD: Substance Use Disorder (ICD10-code: F1) | | | | | | | | |

# **Table S3. Within-individual incident rate ratio of suicidal behaviour in months relative to first SSRI initiation**

|  | **Overall** | | **6 - 17 years** | | **18 - 24 years** | | **25 - 39 years** | | **40 - 49 years** | | **50 - 59 years** | |
| --- | --- | --- | --- | --- | --- | --- | --- | --- | --- | --- | --- | --- |
| **Month** | **No. Events** | **IRR (95% CI)** | **No. Events** | **IRR (95% CI)** | **No. Events** | **IRR (95% CI)** | **No. Events** | **IRR (95% CI)** | **No. Events** | **IRR (95% CI)** | **No. Events** | **IRR (95% CI)** |
| **Reference: month -12** | | | | | | | | | | | | |
| **-12** | 378 | 1 | 49 | 1 | 123 | 1 | 105 | 1 | 58 | 1 | 43 | 1 |
| **-1** | 2,987 | 7.35 (6.60-8.18) | 459 | 9.01 (6.70-12.10) | 921 | 7.07 (5.85-8.53) | 772 | 6.77 (5.52-8.31) | 463 | 7.35 (5.59-9.68) | 372 | 7.73 (5.63-10.62) |
| **+1** | 2,125 | 4.47 (3.99-5.00) | 339 | 6.29 (4.64-8.52) | 705 | 4.45 (3.65-5.43) | 550 | 3.88 (3.12-4.82) | 277 | 3.97 (2.96-5.31) | 254 | 4.56 (3.26-6.39) |
| **+2** | 1,593 | 4.31 (3.84-4.84) | 364 | 7.60 (5.61-10.29) | 567 | 4.71 (3.85-5.75) | 368 | 3.44 (2.75-4.30) | 170 | 3.14 (2.31-4.27) | 124 | 3.08 (2.15-4.40) |
| **+3** | 1,208 | 3.98 (3.54-4.49) | 279 | 6.67 (4.90-9.08) | 437 | 4.43 (3.60-5.44) | 270 | 3.20 (2.54-4.05) | 134 | 3.10 (2.26-4.25) | 88 | 2.59 (1.78-3.76) |
| **+4** | 950 | 4.02 (3.55-4.55) | 243 | 6.77 (4.95-9.26) | 336 | 4.39 (3.55-5.44) | 216 | 3.49 (2.74-4.45) | 87 | 2.68 (1.90-3.77) | 68 | 2.60 (1.76-3.86) |
| **+5** | 681 | 3.50 (3.07-3.99) | 165 | 5.35 (3.86-7.41) | 247 | 4.03 (3.22-5.04) | 144 | 2.84 (2.19-3.69) | 71 | 2.67 (1.86-3.81) | 54 | 2.50 (1.65-3.78) |
| **+6** | 564 | 3.42 (2.99-3.91) | 153 | 5.68 (4.09-7.89) | 197 | 3.81 (3.01-4.81) | 118 | 2.79 (2.12-3.67) | 62 | 2.75 (1.90-3.97) | 34 | 1.89 (1.19-3.01) |
| **+7** | 467 | 3.31 (2.88-3.81) | 114 | 4.75 (3.37-6.70) | 162 | 3.72 (2.91-4.75) | 109 | 3.04 (2.30-4.02) | 67 | 3.66 (2.54-5.27) | 15 | 0.97 (0.53-1.76) |
| **+8** | 351 | 2.89 (2.49-3.36) | 81 | 3.74 (2.60-5.38) | 114 | 3.12 (2.39-4.06) | 94 | 3.14 (2.35-4.20) | 42 | 2.66 (1.76-4.01) | 20 | 1.49 (0.87-2.57) |
| **+9** | 332 | 3.18 (2.73-3.70) | 88 | 4.67 (3.27-6.69) | 113 | 3.59 (2.75-4.68) | 79 | 3.13 (2.31-4.25) | 30 | 2.23 (1.42-3.52) | 22 | 1.86 (1.09-3.15) |
| **+10** | 257 | 2.89 (2.45-3.40) | 76 | 4.59 (3.18-6.64) | 87 | 3.36 (2.52-4.47) | 55 | 2.57 (1.83-3.60) | 21 | 1.83 (1.10-3.06) | 18 | 1.73 (0.98-3.05) |
| **+11** | 237 | 3.06 (2.58-3.62) | 59 | 4.09 (2.77-6.03) | 88 | 3.91 (2.94-5.20) | 56 | 3.05 (2.17-4.28) | 25 | 2.42 (1.49-3.93) | 9 | 0.99 (0.48-2.07) |
| **+12** | 179 | 2.68 (2.23-3.22) | 54 | 4.18 (2.81-6.21) | 65 | 3.46 (2.53-4.73) | 33 | 2.09 (1.39-3.13) | 19 | 2.14 (1.25-3.65) | 8 | 1.01 (0.47-2.17) |
| **Reference: month -1** | | | | | | | | | | | | |
| **-1** | 2,987 | 1 | 459 | 1 | 921 | 1 | 772 | 1 | 463 | 1 | 372 | 1 |
| **+1** | 2,125 | 0.62 (0.58-0.65) | 339 | 0.70 (0.61-0.81) | 705 | 0.64 (0.57-0.71) | 550 | 0.59 (0.52-0.66) | 277 | 0.55 (0.47-0.65) | 254 | 0.60 (0.50-0.71) |
| **+2** | 1,593 | 0.59 (0.56-0.63) | 364 | 0.85 (0.73-0.98) | 567 | 0.67 (0.60-0.75) | 368 | 0.52 (0.45-0.59) | 170 | 0.44 (0.36-0.53) | 124 | 0.41 (0.33-0.50) |
| **+3** | 1,208 | 0.55 (0.51-0.59) | 279 | 0.75 (0.64-0.87) | 437 | 0.63 (0.56-0.71) | 270 | 0.48 (0.41-0.55) | 134 | 0.43 (0.35-0.53) | 88 | 0.34 (0.27-0.44) |
| **+4** | 950 | 0.55 (0.51-0.60) | 243 | 0.76 (0.64-0.89) | 336 | 0.63 (0.55-0.72) | 216 | 0.52 (0.44-0.61) | 87 | 0.37 (0.29-0.47) | 68 | 0.34 (0.26-0.45) |
| **+5** | 681 | 0.48 (0.44-0.53) | 165 | 0.60 (0.50-0.72) | 247 | 0.57 (0.49-0.67) | 144 | 0.42 (0.35-0.51) | 71 | 0.37 (0.29-0.48) | 54 | 0.33 (0.24-0.44) |
| **+6** | 564 | 0.47 (0.43-0.52) | 153 | 0.64 (0.53-0.77) | 197 | 0.54 (0.46-0.64) | 118 | 0.41 (0.34-0.51) | 62 | 0.38 (0.29-0.50) | 34 | 0.25 (0.17-0.36) |
| **+7** | 467 | 0.46 (0.41-0.51) | 114 | 0.53 (0.43-0.66) | 162 | 0.53 (0.44-0.64) | 109 | 0.45 (0.37-0.56) | 67 | 0.51 (0.39-0.67) | 15 | 0.13 (0.07-0.21) |
| **+8** | 351 | 0.40 (0.35-0.45) | 81 | 0.42 (0.33-0.54) | 114 | 0.44 (0.36-0.55) | 94 | 0.47 (0.37-0.59) | 42 | 0.37 (0.27-0.52) | 20 | 0.19 (0.12-0.31) |
| **+9** | 332 | 0.44 (0.39-0.49) | 88 | 0.53 (0.41-0.67) | 113 | 0.51 (0.42-0.63) | 79 | 0.47 (0.37-0.60) | 30 | 0.31 (0.21-0.46) | 22 | 0.24 (0.15-0.38) |
| **+10** | 257 | 0.40 (0.35-0.46) | 76 | 0.52 (0.40-0.67) | 87 | 0.48 (0.38-0.61) | 55 | 0.38 (0.29-0.51) | 21 | 0.26 (0.16-0.40) | 18 | 0.23 (0.14-0.37) |
| **+11** | 237 | 0.42 (0.37-0.49) | 59 | 0.46 (0.35-0.61) | 88 | 0.56 (0.44-0.70) | 56 | 0.46 (0.34-0.61) | 25 | 0.34 (0.22-0.52) | 9 | 0.13 (0.07-0.25) |
| **+12** | 179 | 0.37 (0.32-0.43) | 54 | 0.47 (0.35-0.63) | 65 | 0.50 (0.38-0.65) | 33 | 0.31 (0.22-0.45) | 19 | 0.30 (0.19-0.48) | 8 | 0.13 (0.06-0.27) |

# **Table S4. Within-individual incidence rate ratio of suicidal behaviour in months relative to first SSRI initiation, stratified by sex**

| **Females** | | | **Males** | |
| --- | --- | --- | --- | --- |
| **Month** | **No. Events** | **IRR (95% CI)** | **No. Events** | **IRR (95% CI)** |
| **-1** | 1,882 | 1 | 1,105 | 1 |
| **+1** | 1,322 | 0.61 (0.56-0.65) | 803 | 0.64 (0.58-0.71) |
| **+2** | 1,069 | 0.62 (0.57-0.67) | 524 | 0.55 (0.50-0.62) |
| **+3** | 841 | 0.58 (0.53-0.63) | 367 | 0.49 (0.44-0.56) |
| **+4** | 674 | 0.58 (0.53-0.64) | 276 | 0.49 (0.43-0.57) |
| **+5** | 499 | 0.52 (0.47-0.58) | 182 | 0.40 (0.34-0.48) |
| **+6** | 423 | 0.52 (0.46-0.58) | 141 | 0.38 (0.31-0.45) |
| **+7** | 326 | 0.46 (0.41-0.52) | 141 | 0.45 (0.37-0.54) |
| **+8** | 252 | 0.41 (0.36-0.47) | 99 | 0.38 (0.31-0.47) |
| **+9** | 249 | 0.47 (0.41-0.54) | 83 | 0.37 (0.29-0.47) |
| **+10** | 194 | 0.43 (0.37-0.50) | 63 | 0.33 (0.25-0.43) |
| **+11** | 178 | 0.45 (0.38-0.53) | 59 | 0.36 (0.27-0.47) |
| **+12** | 136 | 0.40 (0.33-0.48) | 43 | 0.31 (0.22-0.42) |

# **Table S5. Within-individual incidence rate ratio of suicidal behaviour relative to first SSRI initiation, stratified by history of suicide attempts and age**

|  | **Overall** | | **6 - 17 years** | | **18 - 24 years** | | **25 - 39 years** | | **40 - 49 years** | | **50 - 59 years** | |
| --- | --- | --- | --- | --- | --- | --- | --- | --- | --- | --- | --- | --- |
| **Month** | **No. Events** | **IRR (95% CI)** | **No. Events** | **IRR (95% CI)** | **No. Events** | **IRR (95% CI)** | **No. Events** | **IRR (95% CI)** | **No. Events** | **IRR (95% CI)** | **No. Events** | **IRR (95% CI)** |
| **With history of suicide attempts prior to month -1 (N = 22,396)** | | | | | | | | | | | | |
| **-1** | 722 | 1 | 99 | 1 | 191 | 1 | 232 | 1 | 115 | 1 | 85 | 1 |
| **+1** | 549 | 0.68 (0.61-0.76) | 71 | 0.69 (0.51-0.94) | 168 | 0.73 (0.59-0.91) | 184 | 0.72 (0.58-0.88) | 74 | 0.60 (0.44-0.81) | 52 | 0.57 (0.40-0.82) |
| **+2** | 346 | 0.56 (0.49-0.64) | 59 | 0.68 (0.49-0.95) | 113 | 0.65 (0.51-0.83) | 106 | 0.54 (0.42-0.69) | 43 | 0.46 (0.32-0.66) | 25 | 0.34 (0.22-0.55) |
| **+3** | 305 | 0.60 (0.52-0.69) | 62 | 0.78 (0.56-1.09) | 106 | 0.76 (0.59-0.98) | 69 | 0.45 (0.34-0.60) | 45 | 0.59 (0.41-0.85) | 23 | 0.36 (0.23-0.59) |
| **+4** | 209 | 0.53 (0.45-0.63) | 47 | 0.68 (0.47-0.97) | 62 | 0.58 (0.43-0.79) | 53 | 0.48 (0.35-0.66) | 27 | 0.48 (0.31-0.75) | 20 | 0.40 (0.24-0.67) |
| **+5** | 180 | 0.57 (0.48-0.67) | 30 | 0.52 (0.34-0.79) | 57 | 0.68 (0.49-0.93) | 50 | 0.57 (0.41-0.79) | 23 | 0.50 (0.31-0.80) | 20 | 0.50 (0.30-0.83) |
| **+6** | 143 | 0.54 (0.44-0.65) | 20 | 0.41 (0.25-0.67) | 44 | 0.62 (0.44-0.88) | 43 | 0.59 (0.42-0.84) | 20 | 0.50 (0.30-0.83) | 16 | 0.48 (0.27-0.84) |
| **+7** | 145 | 0.64 (0.53-0.78) | 34 | 0.77 (0.51-1.16) | 43 | 0.74 (0.52-1.06) | 40 | 0.64 (0.44-0.92) | 21 | 0.65 (0.39-1.07) | 7 | 0.24 (0.11-0.53) |
| **+8** | 99 | 0.51 (0.40-0.63) | 22 | 0.56 (0.34-0.91) | 32 | 0.65 (0.43-0.97) | 29 | 0.53 (0.35-0.81) | 13 | 0.48 (0.26-0.89) | 3 | 0.12 (0.04-0.37) |
| **+9** | 71 | 0.42 (0.33-0.55) | 13 | 0.38 (0.21-0.69) | 27 | 0.65 (0.42-1.00) | 14 | 0.31 (0.18-0.55) | 8 | 0.33 (0.16-0.70) | 9 | 0.40 (0.19-0.82) |
| **+10** | 64 | 0.46 (0.35-0.60) | 15 | 0.52 (0.29-0.92) | 21 | 0.61 (0.37-0.98) | 16 | 0.43 (0.25-0.74) | 6 | 0.30 (0.13-0.69) | 6 | 0.32 (0.14-0.76) |
| **+11** | 57 | 0.46 (0.35-0.62) | 4 | 0.17 (0.06-0.47) | 25 | 0.74 (0.47-1.17) | 15 | 0.47 (0.27-0.81) | 8 | 0.43 (0.20-0.92) | 5 | 0.34 (0.13-0.86) |
| **+12** | 39 | 0.37 (0.27-0.52) | 6 | 0.31 (0.13-0.72) | 16 | 0.55 (0.32-0.95) | 8 | 0.28 (0.14-0.58) | 6 | 0.39 (0.17-0.92) | 3 | 0.24 (0.07-0.78) |
| **Without history of suicide attempts prior to month -1 (N = 516,181)** | | | | | | | | | | | | |
| **-1** | 2,265 | 1 | 360 | 1 | 730 | 1 | 540 | 1 | 348 | 1 | 287 | 1 |
| **+1** | 1,576 | 0.65 (0.60-0.70) | 268 | 0.80 (0.67-0.94) | 537 | 0.66 (0.59-0.75) | 366 | 0.57 (0.49-0.66) | 203 | 0.58 (0.48-0.70) | 202 | 0.65 (0.53-0.80) |
| **+2** | 1,247 | 0.66 (0.61-0.71) | 305 | 1.01 (0.86-1.19) | 454 | 0.73 (0.65-0.83) | 262 | 0.54 (0.46-0.63) | 127 | 0.46 (0.37-0.58) | 99 | 0.47 (0.37-0.60) |
| **+3** | 903 | 0.58 (0.54-0.64) | 217 | 0.83 (0.70-1.00) | 331 | 0.65 (0.56-0.75) | 201 | 0.52 (0.44-0.62) | 89 | 0.41 (0.32-0.53) | 65 | 0.37 (0.28-0.50) |
| **+4** | 741 | 0.61 (0.56-0.67) | 196 | 0.89 (0.74-1.07) | 274 | 0.70 (0.60-0.81) | 163 | 0.57 (0.47-0.69) | 60 | 0.36 (0.27-0.49) | 48 | 0.36 (0.26-0.50) |
| **+5** | 501 | 0.50 (0.45-0.56) | 135 | 0.71 (0.57-0.87) | 190 | 0.60 (0.51-0.72) | 94 | 0.40 (0.32-0.51) | 48 | 0.36 (0.26-0.49) | 34 | 0.31 (0.21-0.45) |
| **+6** | 421 | 0.50 (0.45-0.56) | 133 | 0.79 (0.64-0.98) | 153 | 0.57 (0.48-0.69) | 75 | 0.39 (0.30-0.50) | 42 | 0.37 (0.27-0.52) | 18 | 0.19 (0.12-0.32) |
| **+7** | 322 | 0.45 (0.39-0.50) | 80 | 0.54 (0.41-0.69) | 119 | 0.53 (0.43-0.65) | 69 | 0.43 (0.33-0.56) | 46 | 0.51 (0.37-0.71) | 8 | 0.10 (0.05-0.21) |
| **+8** | 252 | 0.41 (0.35-0.47) | 59 | 0.44 (0.33-0.58) | 82 | 0.43 (0.34-0.55) | 65 | 0.49 (0.37-0.65) | 29 | 0.37 (0.25-0.55) | 17 | 0.25 (0.15-0.42) |
| **+9** | 261 | 0.49 (0.43-0.56) | 75 | 0.64 (0.49-0.84) | 86 | 0.52 (0.41-0.67) | 65 | 0.58 (0.44-0.76) | 22 | 0.34 (0.21-0.53) | 13 | 0.22 (0.12-0.39) |
| **+10** | 193 | 0.42 (0.36-0.49) | 61 | 0.59 (0.44-0.79) | 66 | 0.49 (0.38-0.64) | 39 | 0.4 0(0.29-0.57) | 15 | 0.27 (0.16-0.47) | 12 | 0.22 (0.12-0.41) |
| **+11** | 180 | 0.45 (0.39-0.53) | 55 | 0.60 (0.44-0.81) | 63 | 0.55 (0.42-0.73) | 41 | 0.50 (0.36-0.70) | 17 | 0.34 (0.20-0.57) | 4 | 0.08 (0.03-0.23) |
| **+12** | 140 | 0.41 (0.34-0.49) | 48 | 0.57 (0.41-0.78) | 49 | 0.52 (0.38-0.71) | 25 | 0.36 (0.24-0.55) | 13 | 0.30 (0.17-0.53) | 5 | 0.12 (0.05-0.29) |

# **Table S6. Within-individual incidence rate ratios of suicidal behaviour in initiation analysis using alternative outcome and exposure definitions**

| **Month** | **No. Events** | **IRR (95% CI)** |
| --- | --- | --- |
| **Alternative exposure: SSRI treatment period defined using assumption of one pill per day** | | |
| -1 | 2,987 | 1 |
| +1 | 2,129 | 0.60 (0.57-0.64) |
| +2 | 1,209 | 0.56 (0.52-0.60) |
| +3 | 847 | 0.47 (0.43-0.51) |
| +4 | 603 | 0.47 (0.43-0.52) |
| +5 | 385 | 0.45 (0.40-0.50) |
| +6 | 276 | 0.40 (0.36-0.46) |
| +7 | 221 | 0.41 (0.36-0.48) |
| +8 | 174 | 0.41 (0.35-0.48) |
| +9 | 126 | 0.35 (0.29-0.43) |
| +10 | 99 | 0.34 (0.28-0.42) |
| +11 | 82 | 0.34 (0.27-0.42) |
| +12 | 74 | 0.35 (0.28-0.45) |
| **Alternative outcome: suicidal behaviour of known intent only^a^** | | |
| -1 | 2,755 | 1 |
| +1 | 1,908 | 0.60 (0.56-0.64) |
| +2 | 1,422 | 0.57 (0.53-0.61) |
| +3 | 1,070 | 0.52 (0.49-0.57) |
| +4 | 827 | 0.52 (0.48-0.57) |
| +5 | 583 | 0.44 (0.40-0.49) |
| +6 | 498 | 0.45 (0.41-0.50) |
| +7 | 412 | 0.43 (0.39-0.49) |
| +8 | 299 | 0.37 (0.32-0.41) |
| +9 | 299 | 0.43 (0.37-0.48) |
| +10 | 220 | 0.37 (0.32-0.42) |
| +11 | 210 | 0.40 (0.35-0.47) |
| +12 | 153 | 0.34 (0.29-0.40) |
| ^a^ ICD-10 codes X60-X84 | | |
